# Superficial Cervical Plexus Block for Retro-auricular Mass Excision in a Patient with High Risk of General Anaesthesia: A Case Report

**DOI:** 10.5152/TJAR.2021.21070

**Published:** 2022-04-01

**Authors:** Uğur Peksöz, Fatih Öner, Ali Ahıskalıoğlu

**Affiliations:** 1 Department of Anaesthesiology and Reanimation, School of Medicine, Ataturk University, Erzurum, Turkey; 2 Department of Otolaryngology, Health Science University, Bölge Research and Training Hospital, Erzurum, Turkey

**Keywords:** Bupivacaine, cervical plexus block, interventional ultrasound, lidocaine, lipoma, regional anaesthesia

## Abstract

The surgical procedure of lipomas is performed under local, regional, or general anaesthesia depending on the location, number, and size of the lipoma. Anaesthesia can be achieved with a superficial cervical plexus block in the short-term surgery of soft tissue lesions in the dermatome areas of the lesser occipital nerve and great auricular nerve. In this article, we presented a high-risk patient with comorbid diseases and difficult airway who underwent superficial cervical plexus block for retro-auricular lipoma excision.

**Main Points**

- Regional anaesthesia techniques can be used in patients with comorbid diseases in head and neck surgery.
- The retro-auricular area can be anesthetized with a superficial cervical plexus block.
- Superficial cervical plexus block should be considered for intraoperative anaesthesia and postoperative analgesia in retro-auricular area surgeries.

## Introduction

Lipomas, histologically very similar to mature adipose tissue, are the most common tumors of mesenchymal origin in the human body. They differ from a simple fat tissue accumulation by creating a thin fibrous capsule around them.^1^ Head, neck, shoulder, and back regions are the most common lipoma locations. Although they have usually small in size, they can reach huge sizes due to being asymptomatic. Total surgical excision is generally a sufficient treatment. While recurrence is rarely seen, sarcomatous transformation is much less common.

Surgical procedures are performed using local anaesthesia, regional anaesthesia, or general anaesthesia, depending on the location, size, number of the lipoma, proximity to vital tissues, and the patient’s current clinical condition. General anaesthesia techniques are mainly used in the surgery of large masses.

**Figure 1.**
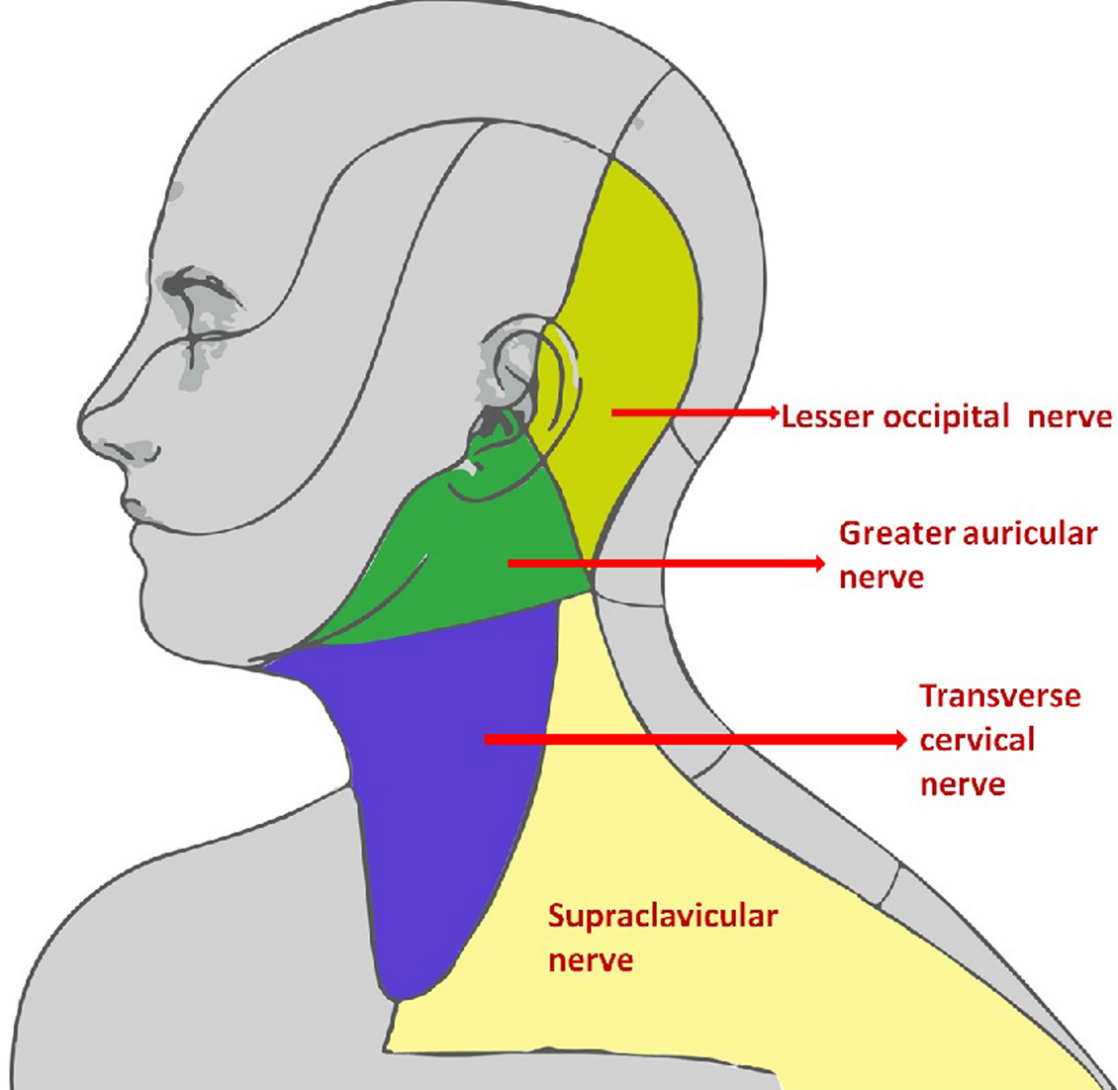
Dermatome areas innervated by the superficial cervical plexus.

The superficial cervical plexus is a network of nerves formed by cervical nerve roots (C2-C4). This network provides significant sensory innervation in the head and neck region; it consists of the transverse cervical nerve, supraclavicular nerve, lesser occipital nerve, and great auricular nerves (Figure 1). Superficial cervical plexus block provides impeding almost all of the superficial branches of the cervical plexus with a single injection.^2^

This case presents the regional anaesthesia technique applied for a short surgical procedure in a morbidly obese patient with comorbid diseases and a difficult airway that prevents general anaesthesia.

## Case Presentation

**Figure 2.**
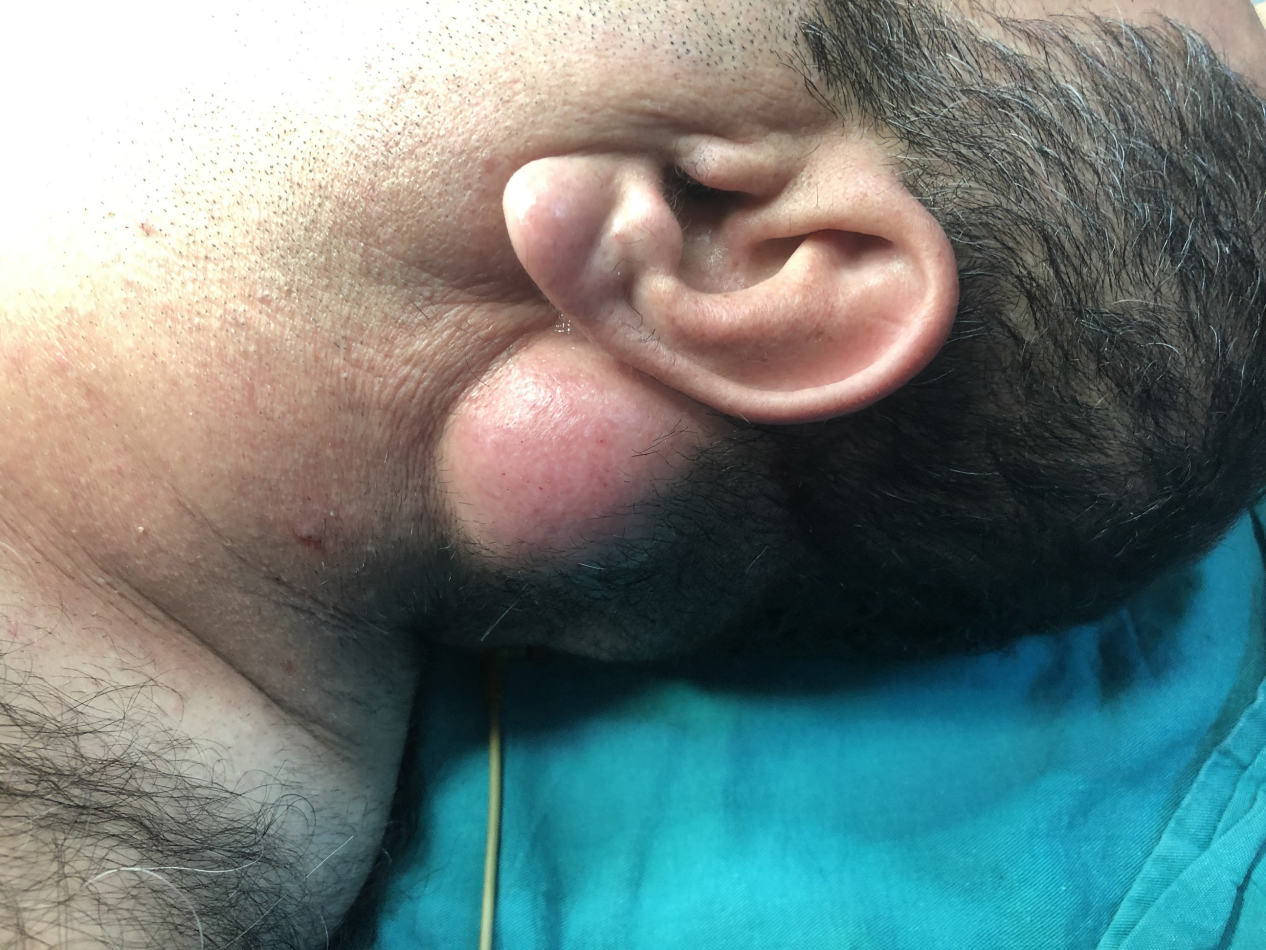
7 × 8 cm lipomatous mass.

A 53-year-old male patient in the American Society of Anesthesiologists III (ASA III) Group with previously known diabetes mellitus, hypertension, and previous cerebral embolism was admitted to the anaesthesia outpatient clinic. The patient had a 7 × 8 cm lipomatous mass behind the left ear and was scheduled for surgery due to intense cosmetic concerns (Figure 2). He was using oral antidiabetic, antihypertensive, and acetylsalicylic acid. The patient, whose body mass index (BMI) was 38 kg m^−2^ (bodyweight: 91 kg, height: 154 cm) had a short and thick neck. Mallampati was graded as class 4. Despite the use of antihypertensive drugs, systemic blood pressure was irregular and high. The mass was located in the lesser occipital nerve and great auricular nerve dermatomes, and we decided to apply a superficial cervical nerve block for surgery. There was no obstacle to regional anaesthesia in his examinations. The patient was informed about the anaesthesia technique and publication and written and verbal consent was obtained.

The patient, placed in a supine position on the operating table, was monitored by the ASA guideline, and appropriate vascular access was provided**. **The patient’s vital signs were as follows: Non-invasive blood pressure: 195/114 mm Hg, pulse: 95 bpm, fingertip SpO_2_: 93% (with 2 L min^−1^ O_2_ support).

To create effective anaesthesia in the surgical field, the block fluid consisting of local anaesthetic agents was prepared in 15 mL with 7.5 mL of 0.5% bupivacaine (Buvasin®, Vem Medical, Tekirdag, Turkey) and 7.5 mL of 2% lidocaine (Aritmal®, Osel Medical, Istanbul, Turkey) in an “anaesthetic concentration” considering the toxic doses. The linear ultrasound (US) probe (Esaote MyLab30®, CA631 high-frequency probe, Cambridge, United Kingdom) was covered sterile. The area was prepared for procedure with 2% chlorhexidine under sterile conditions. Two milligrams midazolam (Sedever®, Haver Medical, Istanbul, Turkey) and 50 µg fentanyl (Fentanyl Citrate®, Hospira, Illionis, USA) were given intravenously to the patient.

**Figure 3.**
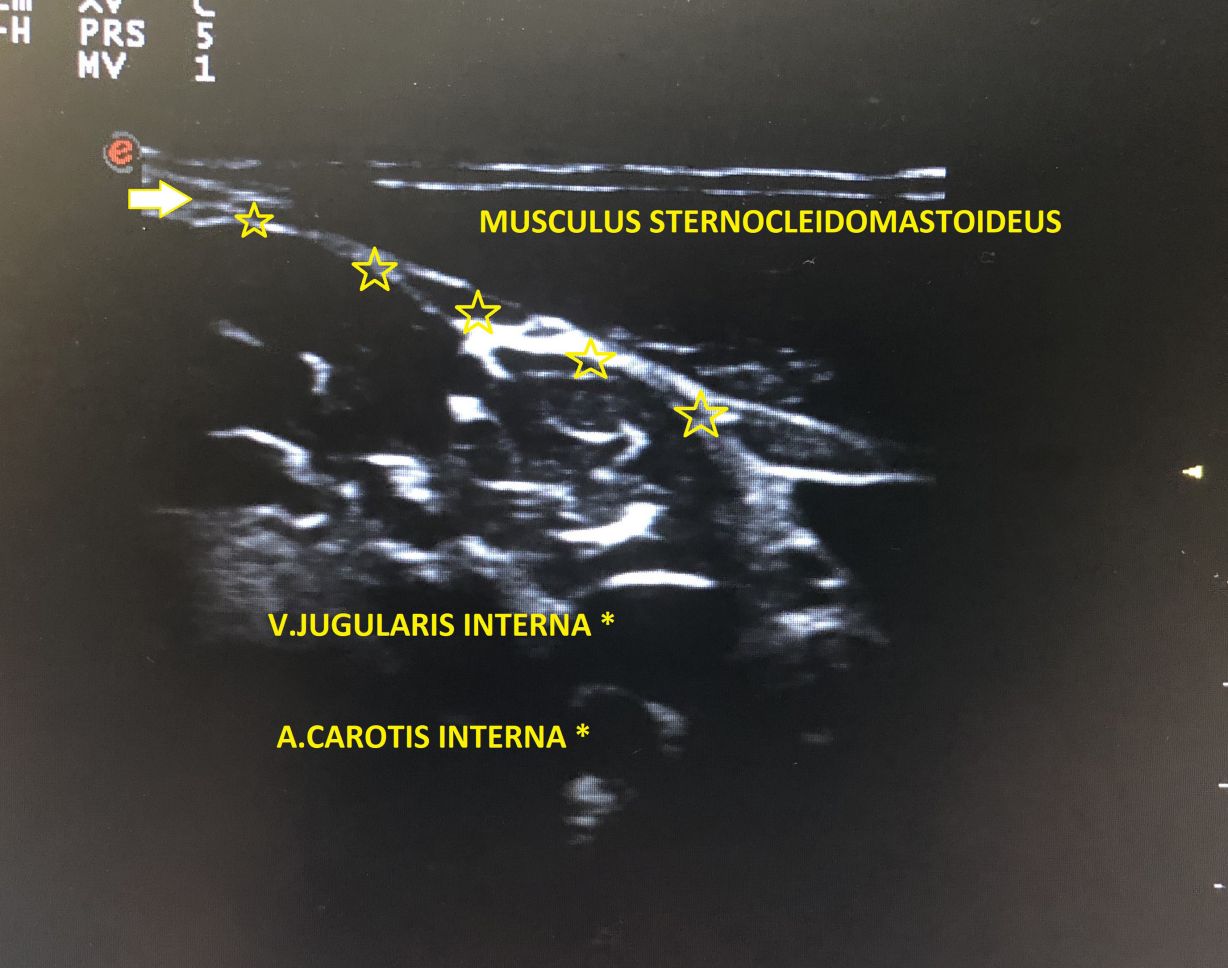
Anatomy under ultrasound. Yellow arrow shows block needle entry point. Yellow stars show local anaesthetic spread plane.

The head of the patient was rotated to the right. The block procedure was performed with the US-guided 50 mm and 20-gauge block needle (Stumplex® Ultra 360®, Braun, Germany) using the in-plane technique, accompanied by instant imaging. While performing the block procedure, the lateral corner of the left sternocleidomastoids muscle at the thyroid cartilage level was visualized by the US. After confirming the injection site with 2 mL 0.9% NaCl, 15 mL of block fluid was injected into the lower part of the muscle’s deep fascia. The diffusion of the drug through the fascia was visualized (Figure 3).

The patient, who could not differentiate between hot and cold in the surgical area dermatomes in the sensory examination performed 15 minutes later with the ice battery, was transferred to surgery. Surgery was successfully completed within 30 minutes.

During surgery, the fluid replacement was performed with the ringer lactate solution. No complications were observed in the patient. The patient did not feel pain during the procedure. After being followed up for a short time in the recovery unit, he was conscious, cooperative, and sent to the clinic without any neurological deficits.

## Discussion

The superficial cervical plexus block is one of the plane blocks. The procedure is performed with local anaesthetic injection into the plan located under the deep fascia of the sternocleidomastoid muscle.^2^ It is successfully used in many operations such as mastoidectomies, carotid endarterectomies, thyroid-parathyroid-larynx, clavicle, and maxillofacial surgeries.^3-5^

General anaesthesia can be performed with a laryngeal mask or intubation, depending on the procedure’s duration. As in this case, the laryngeal mask may not be safe in surgeries requiring a different head position. In patients with difficult airways, difficulties may be encountered in inserting airways.

In this case, general anaesthesia techniques could not be applied because of comorbid diseases, difficult airway, high BMI, and the head’s right rotation. Continuing surgery with local infiltration and sedation was not sufficient and safe for the patient and the surgeon’s comfort due to the patient’s medical problems and the giant lipoma. Since the surgical area is in the lesser occipital nerve and great auricular nerve dermatomes, a superficial cervical plexus block with the US was applied using an anaesthetic dose of local anaesthetic medication.

During the blocking procedure, care should be taken due to risks such as intravascular injection of the local anaesthetic agent, pneumothorax, phrenic nerve block, and vasovagal syncope due to pain. The injection should be followed instantly with the US, and the block needle should not be overlooked.^2^

Maximum attention should be paid to the anaesthesia and vital signs of the patients at every surgery stage. Administration of additional analgesics and sedatives in minimal doses may increase the quality of anaesthesia.

## Conclusion

In the short-term surgery of soft tissue lesions in the lesser occipital nerve and great auricular nerve dermatome areas, anaesthesia can be achieved through superficial cervical plexus block.
